# Merging NMR Data and Computation Facilitates Data-Centered Research

**DOI:** 10.3389/fmolb.2021.817175

**Published:** 2022-01-17

**Authors:** Kumaran Baskaran, D. Levi Craft, Hamid R. Eghbalnia, Michael R. Gryk, Jeffrey C. Hoch, Mark W. Maciejewski, Adam D. Schuyler, Jonathan R. Wedell, Colin W. Wilburn

**Affiliations:** Department of Molecular Biology and Biophysics, UConn Health, Farmington, CT, United States

**Keywords:** data federation, structural biology, data repositories, reproducible research, nuclear magnetic resonance

## Abstract

The Biological Magnetic Resonance Data Bank (BMRB) has served the NMR structural biology community for 40 years, and has been instrumental in the development of many widely-used tools. It fosters the reuse of data resources in structural biology by embodying the FAIR data principles (Findable, Accessible, Inter-operable, and Re-usable). NMRbox is less than a decade old, but complements BMRB by providing NMR software and high-performance computing resources, facilitating the reuse of software resources. BMRB and NMRbox both facilitate reproducible research. NMRbox also fosters the development and deployment of complex meta-software. Combining BMRB and NMRbox helps speed and simplify workflows that utilize BMRB, and enables facile federation of BMRB with other data repositories. Utilization of BMRB and NMRbox in tandem will enable additional advances, such as machine learning, that are poised to become increasingly powerful.

## Introduction and Overview

The Biological Magnetic Resonance Data Bank (Ulrich et al., 2008), BMRB, (bmrb.io) was conceived and launched by John Markley and Eldon Ulrich 40 years ago and has grown to become an indispensable resource for nuclear magnetic resonance (NMR) structural biology, and is emerging as a resource for all aspects of biomolecular NMR, including metabolomics. With the support of the bio-NMR community by depositing data, BMRB now contains over 14,000 curated depositions. The most impactful data hosted by BMRB has been the collection of 9 million assigned ^1^H, ^13^C, ^15^N, and ^31^P chemical shifts for proteins and nucleic acids. The archive continues to grow in size and expand in scope, with increasingly important collections of residual dipolar couplings (RDCs), relaxation data, and hydrogen exchange rates for biomacromolecules. In the near future we anticipate significant depositions of chemical shift tensors from solid-state NMR experiments and empirical time-domain data. The chemical shift archive has been particularly impactful for NMR structural biology, contributing to the development of tools such as TALOS (Shen et al., 2009; Shen and Bax, 2015), SPARTA (Shen and Bax, 2007; Shen and Bax, 2010), SHIFTX (Neal et al., 2003; Han et al., 2011), Chemical Shift Index (CSI) (Wishart and Sykes, 1994; Hafsa and Wishart, 2014; Hafsa et al., 2015), and CS-Rosetta (Shen et al., 2008). These tools relied on broad trends originally discovered in the 1960s (Markley et al., 1967) along with their structural correlates revealed by the chemical shift archive and the federated Protein Data Bank (Burley et al., 2017; Burley et al., 2019) (PDB) archive. The archive has now grown so that extreme chemical shifts previously deemed “outliers” are sufficiently abundant to reveal structural insights (Baskaran et al., 2021). By embodying the FAIR data principles (Findable, Accessible, Interoperable, Reusable) (Wilkinson et al., 2016) BMRB is an essential resource for data science in NMR structural biology.

NMRbox (Maciejewski et al., 2017) (nmrbox.org) is much younger than BMRB, established in 2013. As a repository and platform for executing NMR software, it is a natural complement to BMRB. NMRbox provides hundreds of NMR and structural biology software packages, versioned and captured in virtual machines, creating an archive enabling recapitulation of analyses conducted with older versions of software. It provides substantial computing resources, including servers configured with multiple processors, large complements of random-access memory, and state-of-the-art graphic processing units (GPUs). It also provides substantial enterprise-class data storage, hierarchical to deliver fast access and geo-dispersed to provide very high data security. NMRbox fosters the development and deployment of meta-software packages that require persistent configurations and interoperation of multiple software packages. NMRbox also provides a “data lake,” comprised of versioned instances of important NMR and structural biology databases, connected locally at high speed to the NMRbox computing assets. The choice of providing a data lake, in contrast to a data warehouse, was motivated by its advantages in supporting a large pool of heterogenous data. NMRbox strives to embody many of the FAIR principles for research software (Lamprecht et al., 2020) and helps promote the FAIR principles for research data at the point of data creation rather than at data curation (Gryk et al., 2019).

**FIGURE 1:**
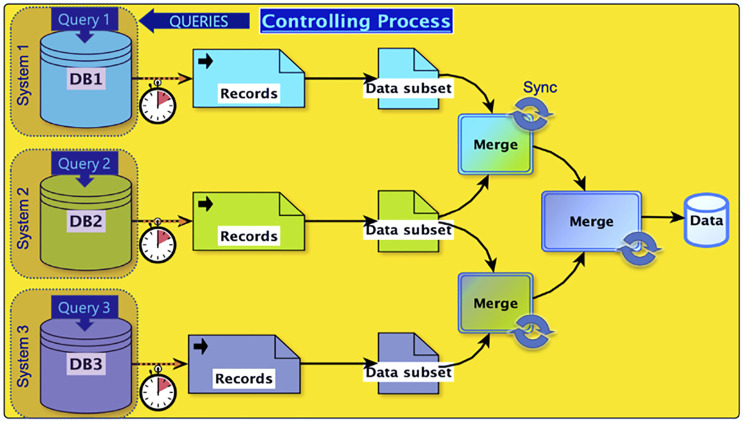
Data flow in a hypothetical study involving federation of three separate databases. An application sends query requests to the three databases. Each database processes the query according to its server load, policies, maintenance cycle, and other factors. Because data records need to be merged, a synchronization step is required. Dotted arrows indicate transfers via the internet. The figure depicts a simple architecture involving 2-way merges. The number of steps and the computational cost of a merge is application-dependent, involving buffer lengths, differential latencies, format conversions, semantic translations, and other factors.

The speed and cost of computation have dramatically improved since the launch of BMRB, and analyses that were once laborious or prohibitive have now become feasible. Improvement in internet connectivity and bandwidth have likewise dramatically improved, fostering federation of BMRB with other data repositories, such as the PDB. Nonetheless data access frequently remains a bottleneck, because the diversity of sources used in a study and the size of relevant databases continue to grow. One approach for mitigating the need for moving large amounts of data is to use the application programming interface (API) for the target data resource and request segments of data only as-needed. Even this approach of distributed queries through the API has limitations, especially when the cumulative amount of data being moved is relatively large. As an example, consider a case where a hypothetical application needs access to records from three different databases (Figure 1). Each database may have its own API, latency, and output format. Thus, the complexity of system-level code for properly synchronizing data receipt and merge operations becomes significant. The ability to complete a complex survey involving this sort of data federation becomes rate limited by the slowest step in a complex set of data transfers, dependent on network latency, bandwidth, and server capacity. Reducing the distance between data and computational resources decrease both the complexity and potential bottlenecks, because repeated movement of data entails costs in both time and resources.

The NMRbox platform provides both compute resources and data resources, thus enabling a straightforward approach to streamlining data federation for structural biology by placing the required data proximal to the computational resources. This has recently been realized by the creation of a “data lake” on NMRbox that houses local snapshots of several important databases for NMR structural biology, notably BMRB, PDB, the NCBI non-redundant sequence database (O'Leary et al., 2016), and most recently the AlphaFold2 database (Jumper et al., 2021) of predicted protein structures for 20 proteomes. This proximity fosters rapid prototyping and development as well as communication between members of a research team. This efficiency has been realized in a recent study of ring current shifts (Baskaran et al., 2021) involving federation of BMRB and PDB entries. By maintaining a copy of each database snapshot in NMRbox, multiple accesses to the same data within the target database is guaranteed to be stable and reproducible.

In this chapter we describe BMRB and NMRbox resources for NMR structural biology, with an emphasis on resources that are less well known and those that take advantage of synergies between the two platforms. These include reference and training resources, resources for group and remote collaboration, and the ReBoxitory data lake. We provide a worked example of a data federation workflow in the form of an interactive Jupyter notebook (https://jupyter.org/).

## NMRbox and Systematized Computation at Scale

### Introduction

**FIGURE 2:**
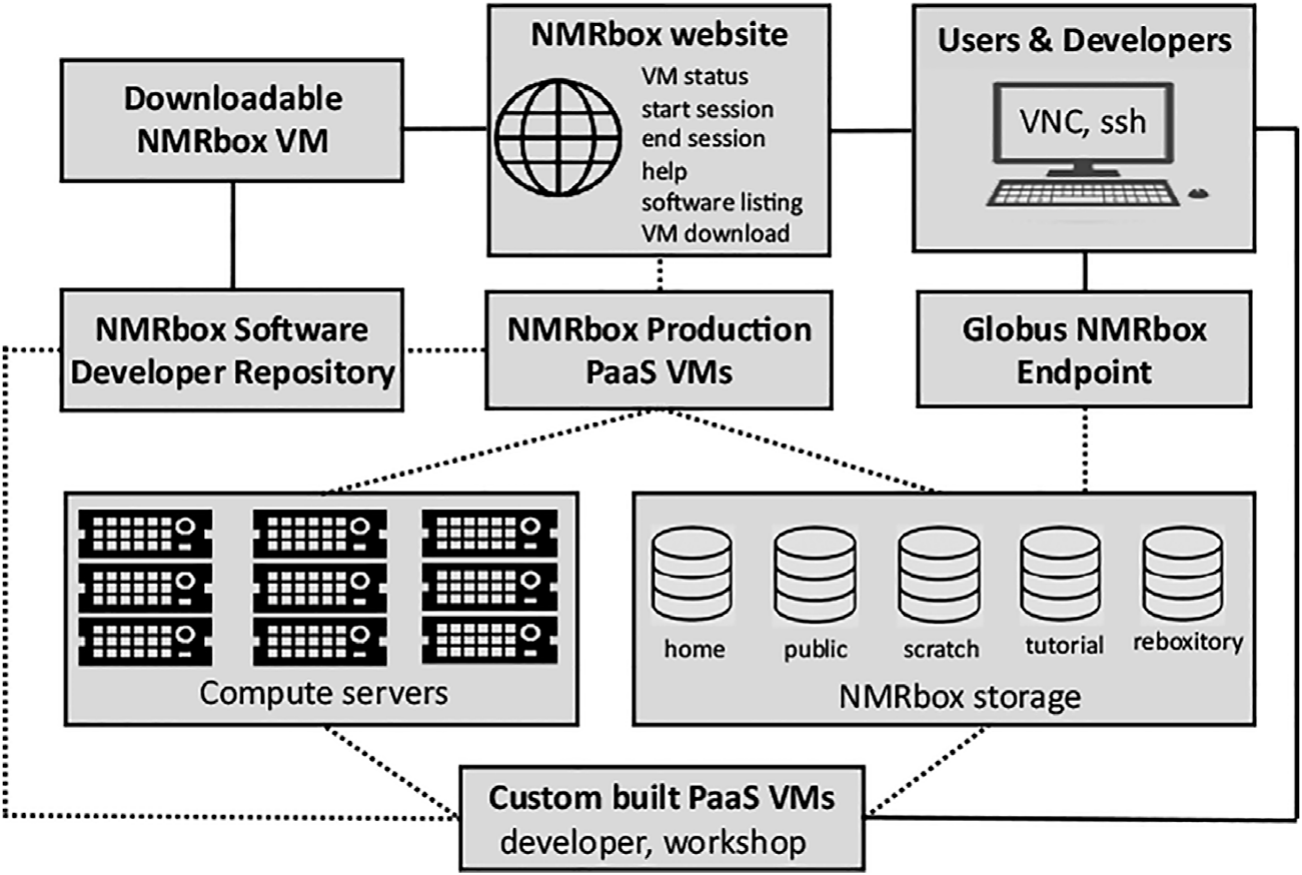
Schematic representation of the NMRbox platform from an end-user and software developer perspective. Solid lines illustrate direct services available to users and developers, while dotted lines represent services that are available transparently through connections controlled by the NMRbox infrastructure. Users connect to NMRbox PaaS VMs (production or custom built) through VNC which provides a full GUI desktop environment or ssh for a command-line interface. The NMRbox website provides users the ability to check the status of VMs, start and end VNC sessions, help documentation, and a complete software listing with version information. File transfers are performed *via* ssh (scp/sftp) or the NMRbox Globus institutional endpoint. All NMRbox VMs (production, custom, and downloadable) are provisioned with software from the NMRbox software developer repository. All PaaS VMs are backed by significant computational resources and storage appliances in the HPC datacenter.

The NMRbox platform (https://nmrbox.org) has been developed by the National Center for Biomolecular NMR Data Processing and Analysis. The Center is a Biomedical Technology Research Resource, supported by the National Institutes of Health/National Institute of General Medical Sciences, grant P41GM111135. The NMRbox platform provides free access to academic, government, and non-profit organizations to a computational platform with hundreds of NMR and related software packages, significant computational and storage resources, and access to significant training materials—all with a near-zero user configuration. To date the platform has 3500 registered users and is used for basic research, workshops, and undergraduate/graduate teaching. A key feature of NMRbox is that it provides software persistence by capturing software in virtual machines (VMs) and maintaining an archive of versioned VMs. This persistence is especial important for software developed in academia by students who move on to other projects. The functional aspects of the NMRbox platform are outlined in Figure 2.

### Scientific Software

NMRbox has taken an agnostic view to installing software packages—we install any software package for which permissions can be acquired and installation is technically feasible within our resources. Software included in NMRbox has been and will continue to be identified through literature searches, BMRB statistics, web-searches, scientific meetings, and user requests. Currently the platform has 200 + software packages covering a wide range of NMR software categories including spectral reconstruction and visualization, automated assignment, structure determination, molecular visualization, validation, chemical shift prediction, dynamics, RDCs, peak picking and analysis, spin simulation, binding analysis, and others. As hybrid approaches become more common, we have expanded our software catalog to include related structural biology software for SAXS (small-angle X-ray scattering), CryoEM (cryo-electron microscopy), molecular dynamics, and bioinformatics tools. The majority of the software packages have been developed in research laboratories, but the NMRbox platform does provide access to some commercial software applications including MNova (Mestrelab Research) and MATLAB (Mathworks Inc.).

### Packaging, Provisioning, and Utility Software

All software included in NMRbox is installed as Debian (an open source Linux distribution) Apt (Advanced package tool) packages from a private NMRbox Apt repository. The Apt packages contain all the files, post and pre installation steps, all system and scientific software dependencies, software metadata information, and can be installed with a simple command making provisioning software in an NMRbox instance straightforward. The entire workflow of Apt package creation is under version control and can be recapitulated if needed. The NMRbox team is attempting to improve upon this process by automating the entire workflow so that as new packages are posted on developers’ sites that NMRbox Apt packages will be automatically rebuilt to reduce the time from a new version being posted to provisioning that new version on the NMRbox platform.

Beyond scientific software applications, the NMRbox platform includes many additional utility and development packages. Included in the platform currently are 15 + text editors, office tools, drawing tools, web-browsers, file transfer tools, fonts, and a variety of helper tools developed by the NMRbox team. In order to aid users in software development efforts we have included many popular programming languages including Python, C, C++, MATLAB, Octave, R, Julia, FORTRAN, Java, Go, PHP, and others along with hundreds of development libraries and tools. The NMRbox team has also authored syntax highlighting for STAR files (Gryk, 2021) (mmCIF and NMR-STAR) which is now included with the standard release of gedit (a simple-to-use and syntax-aware text editor).

### Software Discovery

Users interact with software in NMRbox in many different manners such as a graphical user interface (GUI), command line, script, or from within a programming language such as Python, R, or MATLAB. A consequence of this is that many programs do not lend themselves to a shortcut in a software launcher menu as typical in desktop operating systems. For this reason, we do not provide links to the NMR software packages from the GUI. To aid in software discovery on the NMRbox platform we provide a software registry on the NMRbox website (https://nmrbox.org/software) with the ability to search by category. In addition, inside the NMRbox platform we provide a GUI based Software Explorer which, like the website software page, is searchable. Information about each package is presented along with the files that are executable to help the user discover how to start the software.

Software utilization—NMRbox production instances utilizes monitoring tools that capture all program executions capturing program name, user, and system resources. Data from the monitoring tools is stored in a relational database for easy queries. Software developers can request detailed information about how many times their software has been executed and by how many different users for arbitrary time frames.

### Data Discovery, Curation, and Provenance

NMR data is produced and consumed by a multitude of software tools in various customized data and file formats. The large volume of diverse data, and customized data services similar to ReBoxitory can create a burden for users to explore and examine either their own or external data housed on NMRbox. One of the goals of the NMRbox Center is to provide tools to foster data interoperability (the I in FAIR) and semantic data management. The current approach uses metadata management tools (widgets) built into the NMRbox virtual desktop environment (Gryk, 2019; Gryk, 2021). As NMRbox is built on Xubuntu (a community-maintained Linux platform), the metadata widgets are built using the GNOME technology stack (free and open-source desktop environment) including GTK (free and open-source graphical toolkit).

**FIGURE 3:**
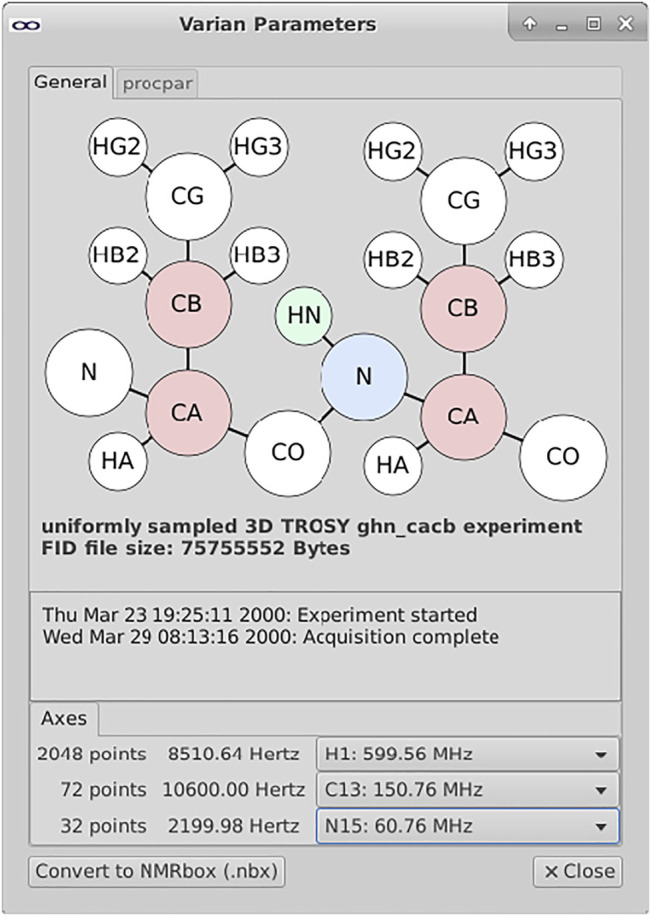
Metadata widget for exploring Varian/Agilent NMR datasets. The metadata widget shown above parses and extracts metadata from the various files within a Varian data directory. This data is used to construct a visual representation of the information layout of the data in terms of the protein atom types correlated along the various spectral dimensions. The mapping between spectral dimensions and axes are not unambiguously defined within the spectrometer metadata; the user is provided with pull-down menus for documenting these relationships.

In the file browser, a right-click on an appropriate file or directory-type invokes an option for displaying the NMR metadata in a graphic window (Figure 3). These windows are designed to include options for searching or browsing the metadata, as well as displaying graphical interpretations or summaries of the data included in the file(s). These widgets also provide the opportunity for users to begin simple curation of their data—for instance, defining which channels of the NMR spectrometer correspond to which dimensions of the NMR experiment. These mappings can be used for translating the data to other formats including an NMRbox data format (.nbx) which includes provenance metadata for eventual deposition to BMRB.

FAIR Data Principle R1.2 requires the recording of metadata/data provenance. That can be quite a challenge for data repositories like BMRB, as if the provenance is not recorded at the time of data creation, it may be impossible to recreate after-the-fact. The NMRbox data format includes a detailed provenance trace of the processing steps which have been applied to a spectral dataset. This provenance metadata is recording using the PREMIS preservation framework which is supported by the Library of Congress (https://www.loc.gov/standards/premis/v3/). This also provides a means for translating the provenance metadata for future inclusion within NMR-STAR entries upon deposition to BMRB (Heintz and Gryk, 2018). Also, as shown by Weatherby and Gryk (2020), the PREMIS provenance record is capable of capturing important metrics/analytics for the data along intermediate steps of the processing pipeline.

### NMRbox Resources

NMRbox computational resources in 2021 provide users with over 75 TFLOPs (trillion floating point operations) of computational power, 14 TB of RAM, 2 TB NVMe, redundant power, redundant 10 GB NICS (network interface controllers), and a NVIDIA T4 GPU for an additional 358 TFLOPs of compute capability. A large expansion of hardware cluster is planned in coming years that will add significant GPU resources, additional CPU cores, and high memory nodes. The NMRbox servers are housed in a state-of-the-art datacenter with backup generator, redundant refrigeration, a 100 GbE (gigabit network) connection to the internet service provider with backup, 40 GbE network fabric in the datacenter, and 80 GbE capable firewalls. An expansion and upgrade of the network capabilities of the datacenter are planned in the coming year to increase capacity and reliability. NMRbox has over 500 TB of network storage on enterprise class storage appliances in addition to 90 TB of ultra-fast NVMe (high-performance random-access memory) local storage. We anticipate that NMRbox resources available to the community will continue to improve as demand grows and technology advances.

NMRbox production instances are deployed as a cloud-based Platform-as-a-Service (PaaS) with high core (36–128 cores) and high memory (192–768 GB) each equipped with an NVIDIA GPU (T4 or A100) for CUDA (*de facto* standard API for GPU computing) processing and accelerated 3D graphics. The base NMRbox operating system (OS) is Xubuntu which build on Xfce, a stable and lightweight desktop environment which works well for remote access compared to GNOME in the standard Ubuntu release. Production instances are simultaneously accessed by multiple end-users and NMRbox employs some technical strategies which attempt to keep the hosts responsive even under high load. User *home folders* are located on a network file system (NFS) and transparently accessible from all NMRbox instances. While NFS home folders bring great convenience, they do suffer from latency which can slow processes that relay on a high rate of I/O operations. To circumvent this limitation each production instance is equipped with an NVMe SSD (solid-state drive) drive that houses a scratch directory with user home folders where users can temporarily copy their files when fast I/O is needed. NMRbox hosts are accessed *via* ssh with or without X11 graphics or more commonly as a full GUI desktop with RealVNC. RealVNC provides single click access to NMRbox hosts with a full GUI, is fully encrypted, allows sessions to be shared, sessions are persistent, allows local printing, and the RealVNC Viewer is free and runs on all modern computers, tablets, and phones. RealVNC allows dynamic screen resolution changes and NMRbox provides a simple screen resolution changer tool with many standard screen resolution sizes, but also allows the users to create custom screen resolutions, set defaults, and create favorites for easy switching.

Upon request purpose-built instances of NMRbox can be built to accommodate situations where NMRbox production instances are not suitable. Special-built instances of NMRbox are designed to limit user access and to grant elevated privileges where appropriate. These instances are labile in nature and generally only persist as needed. Two common situations for purpose-built instances are software developers attempting to deploy their software on the NMRbox platform and workshops where resources can be limited only to workshop participants and instances can be modified readily.

The majority of users access NMRbox as a PaaS, but NMRbox offers a downloadable virtual machine (VM). Initially the downloadable VM provided almost all the software in the PaaS version, but as the number of software offering increased the size of the VM became too large for many standard computers. The downloadable VM now comes as a lightweight base version of NMRbox and an NMRbox software installation tool. Users select which of the software packages from NMRbox that they want to install and can control when software upgrades occur. It should be noted that while most of the software packages are available on the downloadable VM, many features are only available on the PaaS version of NMRbox and the NMRbox team cannot provide extensive support for the different virtualization software used to host downloadable VMs.

### Operations and Services

Governance, user privacy, and licensing. NMRbox data governance policies are designed to support the guidance provided by the National Institutes of Health (NIH) policy statements on data, data sharing, and data ownership. In general, grant recipients own the rights in data resulting from a grant-supported project as described by NIH’s “Rights in Data” statement. To maintain confidentiality, the content of user directories is private and under the control of the user (or research PI). The decision to make the data public, provide additional access, or deposit the data in a public repository is managed by the user. As part of normal administrative operations, NMRbox performs data backups and metadata collection related to computational and resource usage. Additionally, ready-to-use virtual machines are available for download for execution in the user’s local environment. Instructions for the use of virtual machines and the necessary prerequisites, including the hypervisor can be found at: https://nmrbox.org/support/faqs/downloadable. NMRbox maintains a neutral policy on software selection. Operationally, the policy is implemented by provisioning virtual machines with software in order to actively support the community of users so long as applicable licensing terms can be satisfied, and a maintainable and executable version of the software is available. NMRbox provides ample computational resources and does not have a prescribed regulation on the use of computational resources as such. However, use of computational resources is actively monitored to ensure that all users can avail themselves of resources in an equitable manner. Special requests for extended use of computational resources are addressed by the NMRbox leadership team. The current license policy can be found at: https://nmrbox.org/pages/licensing.

Rolling release schedule—In the past NMRbox production instances were upgraded every 6–9 months and all instances were taken off-line together for a few hours while the upgrade was completed. This resulted in 1) all production instances being offline together disrupting users, 2) the time interval between software updates to be too long, 3) users not being able to start long calculations ahead of a shutdown for a new release, and 4) NMRbox instances becoming unstable after 6–9 months of multi-user use. Currently NMRbox employs a rolling release cycle where 1/6th of the NMRbox instances are shut down and rebuilt with a new NMRbox version every 2 weeks. The rolling release cycle eliminates the issues above by allowing; 1) most production instances to remain running even during an upgrade, 2) new or updated software packages to be deployed on production instances quickly, 3) users to select instances with a long time-to-live for long duration calculations, and 4) no instance remains running for more than 12 weeks reducing instability issues. A major goal of NMRbox is the persistence of the NMRbox production instances and older versions of NMRbox instances are archived and many remain running so users can access older software versions as needed. With the new rolling release cycle the first group in each 12-week cycle is listed as a long-term support version which is archived and remains persistent. Older versions remain running, with reduced hardware resources, until security updates end at which point a user needs to request that the old instance be started.

Technical support—NMRbox provides support through a variety of methods including a simple support email, support@nmrbox.org. Support emails are sent to Zendesk support ticketing system where NMRbox agents can respond and track ticket status. We have included a Help Request button inside NMRbox instances, found by clicking the NMRbox icon, allowing easy creation of support tickets. In addition to the support email NMRbox provides self-service support on the NMRbox website through the FAQ page and the Getting Started Guide which defines many of the features and their use on the NMRbox platform. The website allows access to a community wide Slack channel for posing questions to the NMRbox users, promote publications, and requesting software and features. The NMRbox YouTube page provides video tutorials.

File access—NMRbox home folders by default are only readable by the owner, but several mechanisms exist for users to share file access; 1) upon request a group of users can all be placed into the same lab group, 2) a self-service group management tool is available from the Groups tab on the user dashboard of the NMRbox website which will create a shared folder for group access, 3) and a public folder exists for sharing data. NMRbox supports scp, sftp, rsync for file transfers and also provides an NMRbox institutional endpoint for Globus transfers. Beyond user files NMRbox provides a tutorial folder with data from all past NMRbox workshops and a ReBoxitory folder which contains file-based databases from the PDB, BMRB, NCBI, and others for easy access to users from the NMRbox platform.

Reboxitory—with the increasing demand for data-centered research, there is a concomitant demand for resources for rapid access and translation. The new integrated data resource called ReBoxitory is a data lake that provides facile and local access to recent time-stamped copies of the BMRB, PDB, NCBI non-redundant sequence database, AlphaFold, and other databases. Read-only copies of the data repositories are mounted on NMRbox platform-as-a-service VMs and versioned (time-stamped) in order to provide reproducibility and continuity of data-centered research. NMRbox currently maintains separate repository snapshots for each month.

User dashboard—The NMRbox website continues to evolve and provide additional services and functionality—in particular the user dashboard provides many functionalities including; 1) a Status tab listing all production instances along with their status, release, hardware configuration, real-time load, connections, and allows unresponsive sessions to be terminated, 2) a Download tab to download a NMRbox virtual machine locally, 3) a Groups tab for managing lab groups and file sharing, 4) a Profile tab for updating your profile, and 5) a Password tab for password management and resets.

Workshops—NMRbox has built several tools to help individuals and organizations host workshops utilizing the NMRbox platform and many of these features are being developed to be self-service. An event that is, created is posted to the NMRbox website. User registration allows users to be placed into an event group. For each new event a folder is created for presenters to place presentation and tutorial data which then automatically is copied into registered user’s home folder, simplifying the management of files during workshops and tutorials.

## BMRB

### Introduction

NMR spectroscopy is unique among biophysical approaches in its ability to provide a broad range of atomic-level information relevant to the structural, dynamic, and chemical properties of biological macromolecules (Takeuchi et al., 2019). The Biological Magnetic Resonance Data Bank (BMRB) (Seavey et al., 1991; Ulrich et al., 2008; Romero et al., 2020) has for the past 30 years archived the spectral and derived data, including assigned chemical shifts, generated by Nuclear magnetic resonance (NMR) spectroscopy of biological systems. The data in BMRB (bmrb.io) is used by researchers from around the world for compiling refined databases, refining algorithms for back calculating chemical shifts from structure, predicting secondary structure and torsion angles in proteins, automating chemical shift assignment protocols, and computing protein tertiary structure from chemical shifts. The data can be accessed and used individually as well as in aggregate form depending on the need. Other classes of data generated in large quantities by NMR spectroscopists are the NMR constraints used to calculate three-dimensional structures. BMRB has taken on the challenge of cataloging, organizing, and distributing these data in a standard format (NMR-STAR) (Ulrich et al., 2019). In collaboration with the PDB, the CCPN project, and others, this work is beginning to produce results that will provide the NMR community with better tools and the structural biology community with improved quantitative data. In 2006, BMRB has become one of the partners of wwPDB (https://www.wwpdb.org) (Berman et al., 2003; wwPDB Consortium, 2019) and the core archive of NMR spectroscopic data (Berman et al., 2007). BMRB served as one of the deposition sites for wwPDB from 2006 to 2016, before the unified deposition system OneDep (https://deposit.wwpdb.org/deposition/) (Young et al., 2017) took over the global deposition based on geographical location. BMRB is now pushing forward to expand the kinds of data archived to include information relevant to the dynamics and chemistry of biological macromolecules.

### Overview of Data-In Services

**FIGURE 4:**
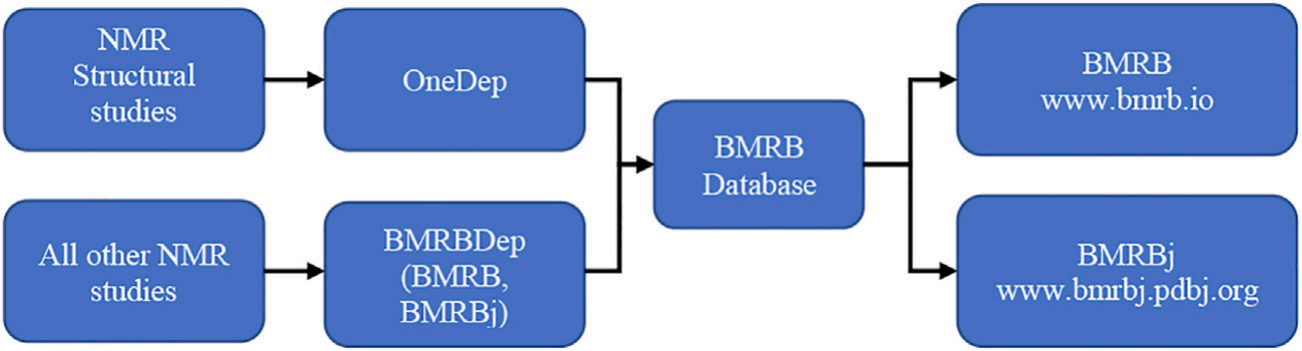
Depending on the study, one of the two modes of BMRB data deposition and dissemination workflow is utilized.

There are two modes of deposition to BMRB (Figure 4). If the NMR study determines a macromolecular structure, then the assigned chemical shifts, restraints and optional NOESY peak list along with the atomic coordinates (structure ensemble) are deposited to wwPDB’s OneDep deposition system (https://deposit.wwpdb.org). The deposition is processed using the OneDep biocuration pipeline (Gore et al., 2017; Young et al., 2017; Young et al., 2018). All other NMR studies can be deposited to either the BMRBDep system running at UConn Health (https://deposit.bmrb.io) or the instance running at BMRBj (https://deposit-bmrb.pdbj.org).

### Data Organization and Maintenance

BMRB has been designed to provide: 1) easy customer access to the data by a number of facilities and in a variety of formats, 2) easy means for data producers to submit data, and 3) a modular architecture that minimizes the impact of individual component updates on the overall system. Data consumers access the Databank through a web browser, an API, or a Globus endpoint for bulk download. Data producers submit data to BMRB either by the OneDep or BMRBdep. In either case, it is possible to incorporate files containing information taken directly from NMR data processing software packages or from databases (including BMRB) as part of the submission. All submissions to the database are processed and returned to the corresponding author for verification before release. After validation and verification, the data are inserted into the BMRB flat-file repository and relational database. BMRB increases the value of the data to customers by adding appropriate links to other databases and information sources.

### Data Dissemination and Content

The NMR spectroscopic data collected though OneDep and BMRBdep deposition system are disseminated to the community in different ways. The coordinate data, assigned chemical shifts and restraints data for structure determination studies can be accessed from all the wwPDB partner sites (RCSB-PDB, PDBe, and PDBj), BRMB and BMRBj. All other NMR data are available in BMRB and BMRBj. BMRB maintains a rich set of NMR-related data including statistical information derived from data for all entries, a variety of reference information, and libraries for user-supplied NMR pulse sequences, software macros, and software. Most data are accepted and exported using the NMR-STAR flat-file format while some are housed in the software formats used when generating the data. BMRB adheres to recognized standards for nomenclature and the reporting of NMR data (Markley et al., 1998). Data standards, formats, and relevant literature references are also posted on the website (https://bmrb.io/standards/). Commercial and academic software vendors are encouraged to provide their customers with tools to graphically display the data contained in BMRB and to export data in formats compatible with BMRB deposition requirements.

Assigned chemical shifts are the most common data and are a requirement for all entries. In addition, several entries contain additional data like distance and dihedral angle restraints, RDCs, coupling constants, relaxation data (*T*_1_/*R*_1_ and *T*_2_/*R*_2_), homonuculear and heteronuclear NOEs, Hydrogen exchange rates, Hydrogen protection factors, Chemical shift Anisotropy (CSA) values and order parameters. The statistics about different data content in different molecules type can be found here (https://bmrb.io/search/query_grid/overview.php).

**FIGURE 5:**
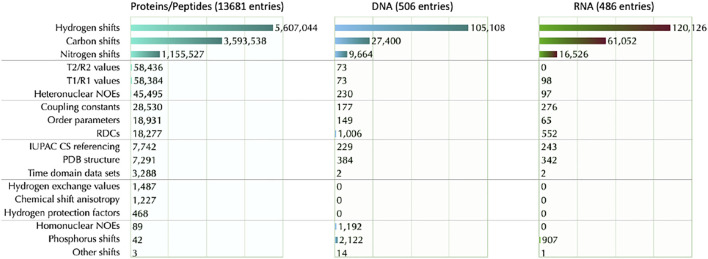
BMRB data content segmented by molecule type and information content. The type of information is segmented into 18 types and indicated along the left side. For each information type, the count of available data is shown to the right of the bar in the bar graph. Data was obtained on date id 2021-W50-4 (specified in ISO week format indicated as year-week-day.

BMRB also provides in-depth statistics for chemical shifts data (https://bmrb.io/ref_info/stats.php)—see Figure 5. The user may choose to have statistics from the full database or from a filtered data set. The filtered data set does not include aromatic and/or paramagnetic ligand(s), or chemical shifts outside eight standard deviations from the average calculated for the full BMRB database, or a chemical shift for at least one carbon bound proton that was greater than 10 ppm or was less than −2.5 ppm. These criteria were used to eliminate from the summary statistics the chemical shifts from paramagnetic proteins, from proteins with aromatic prosthetic groups, and from entries where unusual chemical shift referencing was used. The chemical shift histograms can be viewed by clicking on the distribution histogram icon in the table.

### BMRB Data Standards and Software Resources

BMRB provides data standards and software resources to prepare the NMR data for BMRB and wwPDB depositions.

**Table:** 

BMRB data standards	
Description	Link
Molecular and nomenclature standards	
Atom Nomenclature of amino acids and nucleic acids	https://bmrb.io/referenc/nomenclature/
Amino acids atom nomenclature conversion table	https://bmrb.io/ref_info/atom_nom.tbl
Amino acid pseudo atom nomenclature conversion table	https://bmrb.io/ref_info/pseudoatom_nom.txt
Nucleic acid pseudo atom nomenclature conversion table	https://bmrb.io/ref_info/napseudoatom_nom.txt
Amido acid description	https://bmrb.io/referenc/commonaa.php
Amino acid hydrophobicity table	https://bmrb.io/referenc/hydroph.shtml
Secondary structure propensities	https://bmrb.io/referenc/choufas.shtml
Amino acid codons	https://bmrb.io/referenc/codons.shtml
Amino acid properties	https://bmrb.io/ref_info/aadata.dat
Experimental standards	
Indirect chemical shift referencing	https://bmrb.io/ref_info/cshift.shtml
Random Coil Chemical shifts (Dyson, Wright, and coworkers)	https://bmrb.io/ref_info/pentapeptide.tbl
Random Coil Chemical shifts (Wüthrich and coworkers)	https://bmrb.io/ref_info/wuthrich_chem_shift.txt
Chemical shift index parameters (Wishart and coworkers)	https://bmrb.io/ref_info/csishift.txt
Sequential and medium-range proton distances in polypeptide secondary structures	https://bmrb.io/referenc/noe-table.shtml
Pulse sequence library	https://bmrb.io/tools/choose_pulse_info.php
Data format standards	
NMR-STAR documentation	https://bmrb.io/dictionary/
NMR-STAR dictionary (GitHub)	https://raw.githubusercontent.com/bmrb-io/nmr-star-dictionary/nmr-star-production/NMR-STAR/internal_106_distribution/NMR-STAR.dic
mmCIF documentation	https://mmcif.wwpdb.org/index.html

### Software Resource

Software resources are maintained in BMRB’s official GitHub repository (https://github.com/bmrb-io). This repository provides access to NMR-STAR dictionary, NMR-STAR parser, data visualization tools (PyBMRB, RBMRB) and other software tools to handle NMR data.

**Table:** 

BMRB software resources	
Software resource	Link
NMR-STAR parser	https://github.com/bmrb-io/PyNMRSTAR
BMRB-API	https://github.com/bmrb-io/BMRB-API
Data visualization in Python (PyBMRB)	https://github.com/bmrb-io/PyBMRB
Data visualization in R	https://github.com/bmrb-io/RBMRB

### BMRB Collaborations

BMRB has been collaborating closely with wwPDB partners since 2006 and contributed to the development of the unified deposition and biocuration pipeline OneDep (Young et al., 2017). BMRB is responsible for the development and maintenance of the NMR related biocuration and validation software code base at the wwPDB. BMRB is a member of wwPDB’s NMR-Validation task force (Montelione et al., 2013; Gore et al., 2017) and leading the NMR validation project at wwPDB. It recently developed tools for NMR restraints validation and integrated them into the OneDep system.

In collaboration with the Center for High-Throughput Computing (CHTC), the Open Science Grid (OSG), and the CS-Rosetta team, and as a service to the community, BMRB provides a web interface for submission of chemical-shift-based NMR structure calculation (https://csrosetta.bmrb.io). The service collects user submitted files for structure calculation, checking for validity, submits jobs to a high-performance computing cluster, monitors job progress, and returns the results to the user. To date, this service has performed over 8000 structure calculations for users.

## A Case Study of Bringing Data and Computation Together—Amide Proton Shift Outliers

### Introduction

The availability of the NMRbox infrastructure and resources, including hundreds of commonly-used software packages, offers a unique opportunity for bringing the data closer to the compute power. The gains in processing complex queries involving federation of BMRB and PDB data resources have been realized in a recent study of anomalous amide ^1^H chemical shfits, in which the PDB and BMRB archives were used jointly. Because the NMRbox ReBoxitory maintains stable snapshots of each database, multiple researchers were able to access the same persistent data. BMRB and PDB are continuously updated and are not presently versioned, making it difficult to precisely recapitulate a survey at a later time. Persistence and stability take on added importance for long development cycles where external databases are likely to have asynchronous updates. Combined with the use of notebook technologies available on the NMRbox platform—for example, the Jupyter notebook—team members were able to capture and share the results in a self-contained and reproducible form. Notebooks could also be versioned and used for instructional and training purposes.

### Computational Workflow

**FIGURE 6:**
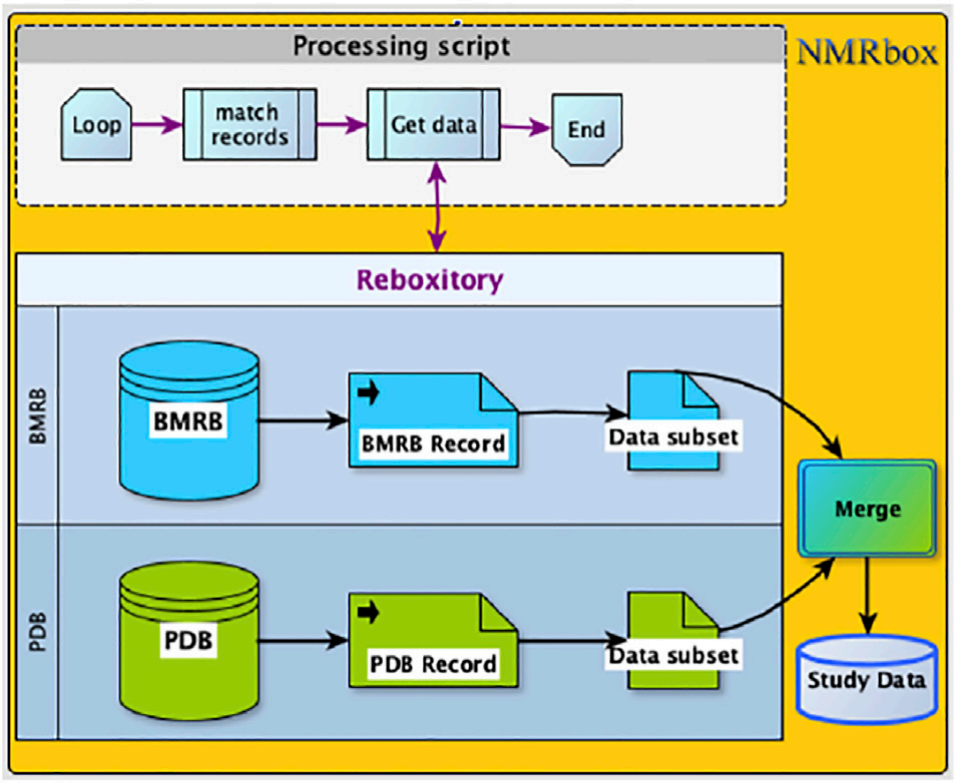
The Processing script and the data are co-located on the NMRbox platform. Access (read), as well data merge (write) operations are managed in a manner that is, similar to the way local files are accessed.

This large-scale study of the role of ring current shifts in anomalously large amide ^1^H shifts required construction of a combined dataset consisting of BMRB chemical shifts and structural data from PDB. The *ReBoxitory* service of NMRbox described earlier provides a highly efficient setting for streamlined data processing by making the PDB and BMRB readily accessible to the processing software. In the ReBoxitory implementation, all references to the data are local and the computation is performed on CPUs within the NMRbox platform (Figure 6). During our study, the proximity of data and computational services accelerated code development and testing. To demonstrate the utility of bringing local repositories close to computational power, we describe two examples implemented via Jupyter notebooks (available online at link).

The Jupyter notebook *amide_aromatic_interaction.ipynb* provides an overall view of the process. The notebook begins by finding the matching BMRB and PDB Ids. This information is available *via* BMRB-API (http://api.bmrb.io/v2/mappings/bmrb/pdb?match_type=exact). Next, the subset of interest comprising amide chemical shifts is extracted from the NMR-STAR formatted entries, and calculations of derived data including a Z-score are performed. Next, coordinate data are extracted from the corresponding CIF entry, and distances are computed. The data sets are merged into a database, using the atom identifiers (sequence id, chain id, residue, and atom). The required computational steps are illustrated by an example notebook (*Demo.ipynb*). Three data classes are created as part of the data merge step—Atom, Residue, and Restraint. The Atom object contains information about its label, the residue it is associated with, and the chemical shift information. The Restraints object maintains information about two Atoms composed of one amide proton and an aromatic ring proton. The code illustrates the steps involving the loading of the BMRB entry from ReBoxitory, parsing the entry information using PyNMRSTAR (see https://github.com/bmrb-io/PyNMRSTAR) and the construction of Residue instances for every residue that will contain Atom instances and for every atom with a reported chemical shift. The restraint file is loaded from the PDB section of ReBoxitory and also parsed using PyNMRSTAR. A search for restraints between amide protons and aromatic ring protons is performed in order to construct a Restraint instance containing two Atom instances.

### Study Design

The purpose of the study was to investigate the connection between amide proton chemical shifts and the potential for hydrogen bonding to an aromatic ring. We searched BMRB for assigned amide protons in proteins corresponding to structures deposited in the PDB. Matching items across databases may require additional steps, but for our study, BMRB provides the list of BMRB and PDB entry id pairs *via* the BMRB API (http://api.bmrb.io/v2/mappings/bmrb/pdb?match_type=exact). As of Jan 2021 we found 7750 BMRB/PDB paired entries and initially retrieved the BMRB entries [in NMR-STAR format (Ulrich et al., 2019)] and PDB entries [in mmCIF format (Bourne et al., 1997)] from their respective databases. We evaluated the data transfer latency by comparing the total remote computation time (remote data access + computation) and the total local computation time (data access + computation). We performed the same survey using the Reboxitory local instances of BMRB and PDB. We found that the average access time for a BMRB-PDB entry pair in the remote configuration is approximately 5.5 s longer. This time difference translated to approximately 8 more hours of total computation time. We prepared a dataset consists of 363686 amide protons from 4670 entries. Finally, during the merge step, we combined the chemical shift information from BMRB and the geometric information form PDB for each amide proton and its nearest aromatic ring, indexed by residue number and name.

The extracted database contained 31,859 amide protons with at least one NOE restraint to a nearby aromatic ring. Once the database of study data was constructed, a Z-score characterizing the deviation of the shift from its mean value from the BMRB database was computed for each assigned amide ^1^H chemical shift. For each assigned amide, the distance from the amide proton position to the center of the nearest aromatic ring was calculated from the coordinates in the PDB mmCIF file. The distance was defined as the average of the distance from the amide proton to the centre of the aromatic ring, averaged over the members of the structural ensemble present in the PDB entry. For the nearest aromatic ring, we calculated an azimuth angle, defined as the angle between a vector normal to the aromatic ring plane and the vector between the amide proton and the center of the ring. The ring normal vector is computed by calculating the cross product of two vectors on the plane of the ring (e.g., the vector from the center of the ring to CG and CD1).

**FIGURE 7:**
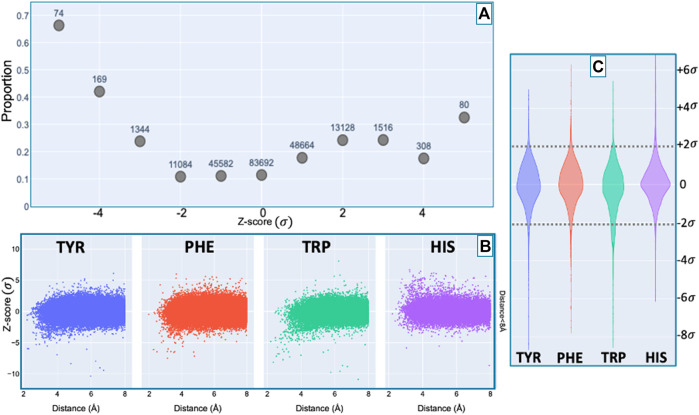
Fraction of amide protons with one or more NOE restraint to an aromatic ring proton (*y*-axis), as a function of the Z-score of the amide proton (*x*-axis) is shown in panel **(A)**. Proportions are calculated with respect to the total number of amide hydrogens with chemical shifts reported in entries with at least one amide-aromatic restraint. The numbers over each point in panel **(A)** are the total number of such amides (including those lacking any NOE restraints to a nearby aromatic) at the corresponding Z-score in units of standard deviation *σ*. The scatter plots in panel **(B)** shows the distribution of amide chemical shifts of amino acids (HIS, TRP, PHE, TYR) as a function of distance (for distances less than 8 Å) of the amide proton from the center of the nearest aromatic ring. For Z-score values between −2σ and 2σ, the restrained amide protons fractions are generally consistent among amino acids studied. However, outside the range of −2σ to 2σ, the trend of restrained amide protons is dependent on the amino acid (HIS, TRP, PHE, TYR). The violin plot in panel **(C)** illustrates the dependence of this trend on the amino acids.

Figure 7 shows the proportion of amide protons exhibiting NOE restraints. In Figure 7B we further demarcate the data by the type of the nearby aromatic residue. For amide protons proximal to Phe, Tyr, or Trp sidechains, there is a noticeable preponderance of extreme upfield shifts (negative Z-score). In contrast, His amide protons exhibiting large deviations from the mean tend to be shifted downfield (positive Z-scores). The upfield-shifted resonances for amides proximal to Phe, Tyr, and Trp are consistent with hydrogen bonding between the amide and the p-π electrons. The downfield-shifted resonances for amides proximal to His are consistent with hydrogen bonding to an electronegative nitrogen atom of the His ring. In-plane downfield ring current shifts are the same sign as the expected downfield shifts arising from hydrogen bonding, with a predicted amide proton ring current shift of 0.5 ppm for an amide nitrogen distance of 3.4 Å. This is consistent with the observation of larger magnitude Z-scores for downfield-shifted amide protons proximal to His. For all four aromatic residue types, there is a clear correlation between proximity to the aromatic ring and the amide chemical shift variance: significant deviations from the mean, corresponding to Z-scores greater than 2, are most likely when the proton is proximal to an aromatic ring, and the magnitude of the shift deviations are larger for closer proximity.

A preponderance of amide-aromatic restraints in upfield-shifted amide protons involved interactions with Trp and Tyr (and to a lesser extent Phe). In contrast, amide protons proximal to His residues predominated strong downfield shifts (Z ≥ 4). For both upfield- and downfield-shifted amide protons, greater deviation from the mean corresponded to the greater the likelihood that corresponding NOE restraints are observed. The trend is noticeably more pronounced for the upfield-shifted amide protons, which is consistent with the formation of hydrogen bonds between the amide and the p-π electrons. The downfield-shifted amides exhibit a weaker correlation, which may be indicative of other dominating effects (not necessarily due to nearby aromatic rings). The data provided corroborating evidence for hydrogen bonding from the amide to the p-π electrons in Trp, Tyr, and Phe, and to the nitrogen atoms in the His ring.

### Discussion and Implications for Future Studies

This preliminary investigation highlights the potential for unlocking latent knowledge hidden in BMRB, PDB, and other biological databases. The challenges posed include curation and validation of the data repositories and federation of data between repositories. Robust and efficient solutions to these challenges are needed in order to realize the full promise of emerging data-centred methods. The recent release of over 300,000 computationally determined protein structures by the DeepMind (Jumper et al., 2021) team can be the first signal indicating the advent of a new era.

AlphaFold is a novel machine learning approach that utilizes physical and biological knowledge about protein structure in combination with leveraging homology models, incorporated in a deep learning algorithm. On initial release, DeepMind has created a repository for 350,000 proteins whose three-dimensional coordinates were predicted using AlphaFold; accessible through https://alphafold.ebi.ac.uk/. With an anticipated 16,000,000 structural predictions by the end of year, it quickly becomes unreasonable to perform certain queries across an online database. For this reason, it has been advantageous to mirror the repository of AlphaFold predictions on NMRbox’s ReBoxitory. An added benefit of a static local repository is to ensure the provenance of data generated using AlphaFold predictions. The significance of this becomes apparent when viewing a AlphaFold coordinate files, which incorporates the version of AlphaFold used to generate the prediction. Having access to these predictions on NMRbox’s suite of virtual machines, in conjunction with its cloud-based computing power, enabled the development of a workflow for predicting the chemical shifts of AlphaFold’s theoretical protein coordinates.

An initial case study of 2,338 proteins were used to implement this workflow. This workflow comprised of querying the ReBoxitory for structure coordinate files, adding hydrogen atoms using a single software package, generating chemical shift predictions using 8 competing computational methods, and depositing the protonated coordinates and chemical shifts in a PostgreSQL database (http://postgresql.org). Even this constrained case study of 18,704 predictions would not have been feasible without the extensive computing power provided by NMRbox. Predictions for the 2,338 proteins using a single chemical shift predictor varied from as little as 10 h to as much as 3 million computing hours if performed on a single CPU. It was imperative high-throughput computing be implemented to address this otherwise insurmountable computational expense. For this reason, HTCondor (Chervenak et al., 2007) was implemented in this workflow to distribute the chemical predictions across the 920 available CPUs in the NMRbox machine pool. This initial case study of this workflow was developed using Common Workflow Language (CWL) (Crusoe et al., 2021), calling upon HTCondor to perform the chemical shift predictions. However, for future studies into investigating the accuracy of different chemical shift predictions and protonation methods, HTCondor directed acyclic graphs, DAGs, will be implemented. This decision was the result of a problem with the reference implementation of CWL not handling job failures in a non-catastrophic manner. This will be mended with HTCondor’s ability to deliver a versatile job management system that will attempt to rerun erroneous actors, as well as error handling to continue the remainder of a workflow and later reporting directly on raised exceptions. However, it is important to note that many of the academic software packages for calculating chemical shifts were not designed for the purpose of high-throughput computation and may continue to present challenges when used in this manner. Experience gained in designing workflows and relational databases will serve the ongoing expansion of predicting the chemical shifts for all AlphaFold predictions, including comparing versions of predictions.

## Conclusion

Facile access to high quality data resources for NMR structural biology such as BMRB and PDB has enabled notable advances, and the growth in the amount and diversity of the data heralds further advances. Increasing federation among data resources and powerful computational approaches, including advanced statistical and machine learning approaches, will further extend the reach of existing resources, unlocking latent insights. The NMRbox platform complements BMRB and PDB by realizing FAIR principles for software, and creating an environment for developing meta-software and supporting complex workflows. The combination of BMRB and NMRbox, as illustrated in the study of ring current shifts for amide proton ^1^H resonances provide a vivid example of how marrying data and computation accelerates discovery. We anticipate that NMR scientists will discover new and important ways to leverage BMRB and NMRbox to address important problems in structural biology.

## Data Availability

Publicly available datasets were analyzed in this study. This data can be found here: bmrb.io and rcsb.org. Access to the NMRbox data lake Reboxitory requires an NMRbox account (nmrbox.org), which is free for academic and not-for-profit users. The Python notebooks used in the amide chemical shift study are located at DOI: 10.5281/zenodo.5703174.
